# Cortical somatosensory evoked potential amplitudes and clinical outcome after cardiac arrest: a retrospective multicenter study

**DOI:** 10.1007/s00415-023-11951-4

**Published:** 2023-08-28

**Authors:** Noelle Aalberts, Erik Westhall, Birger Johnsen, Katrin Hahn, Martin Kenda, Tobias Cronberg, Hans Friberg, Sandra Preuß, Christoph J. Ploner, Christian Storm, Jens Nee, Christoph Leithner, Christian Endisch

**Affiliations:** 1 Department of Neurology, AG Emergency and Critical Care Neurology, Campus Virchow-Klinikum, Charité Universitätsmedizin Berlin, Augustenburger Platz 1, 13353 Berlin, Germany; 2 Department of Clinical Sciences Lund, Clinical Neurophysiology, Lund University, Skane University Hospital, Getingevägen 4, 22185 Lund, Sweden; 3 Department of Clinical Neurophysiology, Aarhus University Hospital and Department of Clinical Medicine, Aarhus University, Palle Juul-Jensens Boulevard 165, 8200 Aarhus N, Denmark; 4 Department of Neurology, Campus Mitte, Charité Universitätsmedizin Berlin, Charitéplatz 1, 10117 Berlin, Germany; 5 BIH Charité Junior Digital Clinician Scientist Program, BIH Biomedical Innovation Academy, Berlin Institute of Health at Charité–Universitätsmedizin Berlin, Charitéplatz 1, 10117 Berlin, Germany; 6 Department of Clinical Sciences Lund, Neurology, Lund University, Skane University Hospital, Getingevägen 4, 22185 Lund, Sweden; 7 Department of Clinical Sciences Lund, Intensive and Perioperative Care, Lund University, Skane University Hospital, Getingevägen 4, 22185 Lund, Sweden; 8 Department of Nephrology and Intensive Care Medicine, Cardiac Arrest Center of Excellence Berlin, Campus Virchow-Klinikum, Charité Universitätsmedizin Berlin, Augustenburger Platz 1, 13353 Berlin, Germany

**Keywords:** Somatosensory evoked potentials, Cardiac arrest, Prognosis, Hypoxic encephalopathy, Coma

## Abstract

**Objective:**

Bilaterally absent cortical somatosensory evoked potentials (SSEPs) reliably predict poor outcome in comatose cardiac arrest (CA) patients. Cortical SSEP amplitudes are a recent prognostic extension; however, amplitude thresholds, inter-recording, and inter-rater agreement remain uncertain.

**Methods:**

In a retrospective multicenter cohort study, we determined cortical SSEP amplitudes of comatose CA patients using a standardized evaluation pathway. We studied inter-recording agreement in repeated SSEPs and inter-rater agreement by four raters independently determining 100 cortical SSEP amplitudes. Primary outcome was assessed using the cerebral performance category (CPC) upon intensive care unit discharge dichotomized into good (CPC 1–3) and poor outcome (CPC 4–5).

**Results:**

Of 706 patients with SSEPs with median 3 days after CA, 277 (39.2%) had good and 429 (60.8%) poor outcome. Of patients with bilaterally absent cortical SSEPs, one (0.8%) survived with CPC 3 and 130 (99.2%) had poor outcome. Otherwise, the lowest cortical SSEP amplitude in good outcome patients was 0.5 µV. 184 (42.9%) of 429 poor outcome patients had lower cortical SSEP amplitudes. In 106 repeated SSEPs, there were 6 (5.7%) with prognostication-relevant changes in SSEP categories. Following a standardized evaluation pathway, inter-rater agreement was almost perfect with a Fleiss’ kappa of 0.88.

**Interpretation:**

Bilaterally absent and cortical SSEP amplitudes below 0.5 µV predicted poor outcome with high specificity. A standardized evaluation pathway provided high inter-rater and inter-recording agreement. Regain of consciousness in patients with bilaterally absent cortical SSEPs rarely occurs. High-amplitude cortical SSEP amplitudes likely indicate the absence of severe brain injury.

**Supplementary Information:**

The online version contains supplementary material available at 10.1007/s00415-023-11951-4.

## Introduction

In multimodal neuroprognostication after cardiac arrest (CA), cortical somatosensory evoked potentials (SSEP) are recommended to predict the severity of hypoxic-ischemic encephalopathy (HIE) [1, 2]. Bilaterally absent cortical SSEPs reliably predict poor outcome in comatose patients [3, 4]. Cortical SSEP amplitudes are a recent prognostic extension beyond the dichotomy of classifying SSEPs as bilaterally present or absent [5–8].

In few studies, low-amplitude cortical SSEP amplitudes predicted poor outcome, whereas high-amplitude cortical SSEP amplitudes indicated absence of severe HIE [5–12]. The prognostic thresholds of cortical SSEP amplitudes varied among these single-center studies. Potential explanations were different methods of amplitude determination, noise levels [3, 13, 14], inter-rater disagreement [15, 16], and cohort differences. Importantly, insufficient recording quality can cause false classification of SSEPs as bilaterally absent in comatose patients without HIE [17] with risk of a self-fulfilling prophecy and decreasing the high specificity of SSEPs in predicting poor outcome [3, 13].

To further investigate the validity of cortical SSEP amplitude as a prognostic test in comatose CA patients, we conducted a large, retrospective, multicenter study with focus on a standardized SSEP evaluation, inter-recording agreement, and inter-rater agreement.

## Methods

### Standard protocol approvals, registrations, and patient consents

Local ethics committees fully approved this study and waived the need for patient consent (EA4/004/14). The study was conducted according to the Declaration of Helsinki.

### Participants

We retrospectively enrolled adult comatose CA patient investigated with median nerve SSEP in four academic centers (Center 1: Charité University Hospital, Berlin, Germany, Campus Virchow-Klinikum, January 2015–December 2019; Center 2: Charité University Hospital, Berlin, Germany, Campus Mitte, December 2011–November 2015; Center 3: Skåne University Hospital, Lund, Sweden, April 2007–April 2016; and Center 4: Aarhus University Hospital, Aarhus, Denmark, September 2010–May 2016). Local postresuscitation care protocols adhered to international guidelines [18, 19]. This included withdrawal of life-sustaining therapy (WLST) based on multimodal neuroprognostication. Bilaterally absent cortical SSEP were used as one parameter within a multimodal approach to predict poor outcome in all centers, whereas only center 1 used cortical SSEP amplitudes above 2.5 µV as a parameter for absence of severe HIE [6]. In center 3, SSEPs were used more selectively [20]. Demographics reporting followed the revised Utstein-style [21].

### SSEP recordings

We placed electrodes at CP3/CP4, C7 (cervical, N13) and over the supraclavicular fossa (NErb) following the international 10–20 system and used a midfrontal (Fz) cortical reference electrode [22, 23]. To reduce noise, we kept skin–electrode impedance below 5 kOhm, and used muscle relaxants and/or sedative bolus if necessary. We recorded 50 ms post-stimulus and averaged at least two 500 repetitions per side. Supplementary Table 1 provides more technical details. Blinded for clinical data, digitalized SSEPs were analyzed using custom-written MATLAB scripts (release 2019b, MathWorks Inc) following a standardized evaluation pathway [6] (Supplementary Fig. 1 and Supplementary Fig. 2). SSEPs were excluded when cervical SSEPs were not bilaterally reproducible (with the exception of reproducible cortical SSEP amplitudes larger than 1.0 µV despite non-reproducible cervical SSEPs) or when noise levels impeded interpretation of cortical SSEPs. Noise level was defined as difference between N_max_ and P_min_ within 5–10 ms after stimulus. Only when noise level was below 0.25 µV in all cortical recordings and cortical potentials were not reproducible, SSEPs were classified as bilaterally absent. For reproducible cortical potentials (at least 4.5 ms after the N13), we determined cortical amplitudes of baseline–N1, N1–P1, baseline–P1, and N_max_–P_min_ in atypical SSEP morphology. The cortical SSEP amplitude was defined as the highest amplitude of all recordings. Amplitudes were rounded to two decimal places.

In patients with repeated SSEPs, we evaluated the second cortical SSEP amplitude blinded for the results of the first SSEP. Based on previous own studies [6, 24, 25], we categorized SSEPs into excluded, bilaterally absent, up to 0.5 µV, larger than 0.5–2.5 µV, and above 2.5 µV. We analyzed the inter-recording agreement between first and second SSEP, and thoroughly reviewed cases with prognostication-relevant amplitude changes.

Furthermore, we studied inter-rater agreement on determination of cortical SSEP amplitudes. Three raters with different neurophysiological expertise (C.L. with more than 10 years; M.K. and N.A. with less than 3 years) underwent a training session with supervisor C.E. (rated all SSEPs, 10 years expertise) on 20 patients with typical SSEP morphologies (Supplementary Fig. 6) following the standardized evaluation pathway (Supplementary Fig. 1 and Supplementary Fig. 2). Subsequently, each rater independently evaluated the last 100 SSEPs not included in the training session, blinded to clinical data and the results of the other raters.

### Outcome

We assessed clinical outcome as primary outcome using the Cerebral Performance Category (CPC) scale upon intensive care unit (ICU) discharge, and classified CPC 1–3 as good and CPC 4–5 as poor outcome. To investigate potential confounders of coma, center specifics in order of prognostic testing, and ICU duration, we reviewed patients with CPC 4 and cortical SSEP amplitudes above 2.5 µV [6, 24, 25]. Furthermore, we assessed from clinical records whether patients regained consciousness, i.e., were awake and communicating during the ICU stay.

### Statistical analysis

Baseline demographics are presented as medians with inter-quartile range (IQR) or absolute numbers with percentage as appropriate. To illustrate the association between cortical SSEP amplitude and investigated parameters, we used scatter plots, and calculated median and IQRs. We used the Kolmogorov–Smirnov test to check for normal distribution and a two-sided Wilcoxon rank-sum test to compare groups as appropriate. Sensitivity and specificity were calculated for outcome prediction. A heatmap illustrates the inter-recording agreement. To analyze inter-rater agreement, SSEPs were categorized into excluded, bilaterally absent, up to 0.5 µV, larger than 0.5–2.5 µV, and above 2.5 µV; and agreement between the four raters was described numerically. Fleiss’s kappa was calculated according to previous conventions [26] as follows: 0–0.20, slight; 0.21–0.40, fair; 0.41–0.60, moderate; 0.61–0.80, substantial; and 0.81–1.00, almost perfect inter-rater agreement. A two-sided p-value below 0.05 was considered statistically significant. All analyses were performed with MATLAB.

### Data availability

Anonymized data are available on reasonable request to the corresponding author.

## Results

### Patient characteristics

We enrolled 816 patients of whom 9 (1.1%) had incomplete recordings, 48 (5.9%) bilaterally non-reproducible cervical potentials with low-amplitude cortical SSEP amplitudes, and 53 (6.5%) patients had too high noise levels. Supplementary Table 2 and Supplementary Table 3 shows the patient flow for the standardized evaluation pathway stratified by each center. Table 1 provides the baseline demographics. Of 706 (85.5%) included patients, 277 (39.2%) had good and 429 (60.8%) poor outcome. Median ICU duration was 10 days (6–21) and 104 (15%) had CPC 4. Median timing of the first SSEP was 3 days (IQR 2–4) after CA. While age (median 62–66 years) and gender distribution (67–81% male) were similar in all centers, other demographic variables relevantly differed. In center 3, 85 patients had the longest median resuscitations (27 min, 15–40), lowest rate of regain of consciousness (4 of 85 patients), and highest WLST rate (74 of 85 patients). 144 patients of center 4 had the highest rate of CPC 4 (38%) with a median ICU duration of 6 days (4–10).

**Table 1:** Baseline characteristics of included patients

	All	Center 1	Center 2	Center 3	Center 4
Patients, *n* (%)	706 (100)	420 (60)	57 (8)	85 (12)	144 (20)
Age, median (IQR), years	64 (52–72)	62 (51–73)	65 (55–73)	66 (53–71)	65 (56–72)
Gender, *n* (%), male	532 (75)	317 (75)	41 (72)	57 (67)	117 (81)
OHCA, %	71	65	51	80	90
Cardiac cause, %	56	50	60	57	77
Shockable rhythm, %	46	40	35	51	67
tROSC, median (IQR), min	18 (10–30)	15 (10–30)	15 (10–30)	27 (15–40)	20 (12–30)
Regain of consciousness, *n* (%)	306 (43)	219 (52)	25 (44)	4 (5)	58 (40)
WLST, *n* (%)	281 (40)	157 (37)	18 (32)	74 (87)	32 (22)
Length of ICU stay, median (IQR), d	10 (6–21)	13 (7–23)	26 (17–35)	6 (4–10)	6 (4–10)
Time until 1. SSEP, median (IQR), d	3 (2–4)	3 (3–4)	5 (3–7)	4 (3–5)	2 (1–4)
Time until 2. SSEP, median (IQR), d	6 (4–7)	7 (6–8)	7 (6–12)	7 (6–11)	2 (2–3)
Outcome upon ICU discharge					
CPC 1, *n* (%)	114 (16)	89 (21)	4 (7)	0 (0)	21 (15)
CPC 2, *n* (%)	108 (15)	72 (17)	11 (19)	2 (2)	23 (16)
CPC 3, *n* (%)	55 (8)	34 (8)	7 (12)	2 (2)	12 (8)
CPC 4, *n* (%)	104 (15)	30 (7)	15 (26)	4 (5)	55 (38)
CPC 5, *n* (%)	325 (46)	195 (46)	20 (35)	77 (91)	33 (23)

Center 1: Charité University Hospital, Berlin, Germany, Campus Virchow-Klinikum; Center 2: Charité University Hospital, Berlin, Germany, Campus Mitte; Center 3: Skåne University Hospital, Lund, Sweden; and Center 4: Aarhus University Hospital, Aarhus, Denmark

*IQR* inter-quartile range, OHCA out-of-hospital cardiac arrest, *tROSC* time from cardiac arrest to spontaneous circulation, *WLST* withdrawal of life-sustaining treatment, *ICU* intensive care unit, *SSEP* somatosensory evoked potential, *CPC* Cerebral performance category

### Cortical SSEP amplitudes and clinical outcome

**Fig. 1:**
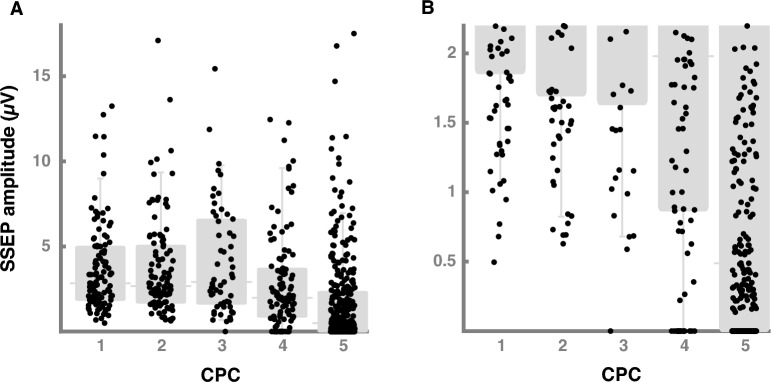
Cortical SSEP amplitudes and clinical outcome. **A** This figure shows the association between cortical SSEP amplitudes and clinical outcome assessed by CPC. We present the results as scatterplots (black dots) and box plots (gray) with inter-quartile range, median and whisker bars representing the 5th and 95th percentile. **B** The y-axis is restricted to the lower cortical SSEP amplitudes to show the lower threshold of CPC 1–3 patients. SSEP somatosensory evoked potential, CPC Cerebral performance category

Figure 1 shows the association between cortical SSEP amplitude and clinical outcome. Median cortical SSEP amplitude was 2.8 µV (1.9–5.0) in CPC 1, 2.7 µV (1.7–5.0) in CPC 2, 2.9 µV (1.6–6.6) in CPC 3, 2.0 µV (0.9–3.7) in CPC 4, and 0.5 µV (0–2.3) in CPC 5 patients.

Of 277 patients with good outcome, one patient with CPC 3 had bilaterally absent cortical SSEP in a first SSEP and 0.51 µV in a repeated SSEP. This was an 18-year-old male who regained consciousness, but suffered from a severe spastic tetraparesis, dysphagia, anarthria, generalized dystonia, and severe cognitive deficits. He did not improve in a 13 months follow-up. Except for this one patient, all other 130 (99.2%) patients with bilaterally absent cortical SSEP had CPC 4 (*n* = 16) or CPC 5 (*n* = 114) yielding a sensitivity of 30.3% to predict poor outcome. Presence of cortical SSEPs yielded a positive predictive value of 48.0% (276/575) to predict good outcome. The lowest cortical SSEP amplitude of CPC 1 patients was 0.50 µV and 0.63 µV in CPC 2 patients. Of 429 patients with CPC 4–5, 184 (42.9%) had cortical SSEP amplitudes below 0.50 µV.

Across centers (Supplementary Fig. 3), range of median cortical SSEP amplitude were 2.38–5.59 µV in CPC 1–3, 0.87–3.11 µV in CPC 4, and 0.25–1.05 µV in CPC 5 patients, respectively. Among CPC 1–3 patients, the lowest cortical SSEP amplitudes was 1.02 µV in center 2, 1.25 µV in center 3, 0.78 µV in center 4, and bilaterally absent and 0.50 µV, respectively, in center 1.

In 306 (43.3%) patients who regained consciousness, median cortical SSEP amplitude was 2.8 µV (1.8–5.4) compared to 0.7 µV (0–2.5, *p* < 0.001) in 400 (56.7%) patients without regain of consciousness. In center 4, 86 patients without regain of consciousness had higher cortical SSEP amplitudes compared to other centers (median 2.3 µV, 0.4–4.5). 58 patients, who regained consciousness in center 4, had significantly higher cortical SSEP amplitudes (median 5.5 µV, 3.7–7.6) compared to 219 patients of center 1 (median 2.5 µV, 1.6–3.7, *p* < 0.001).

20 of 131 patients (6.5%) with regain of consciousness had cortical SSEP amplitudes below 1 µV, 176 (57.5%) above 2.5 µV, and 85 (27.8%) above 5 µV. The positive predictive value for a threshold of 2.5 and 5 µV to predict regain of consciousness was 64.0% (176/275) and 68.6% (85/124), respectively.

**Fig. 2:**
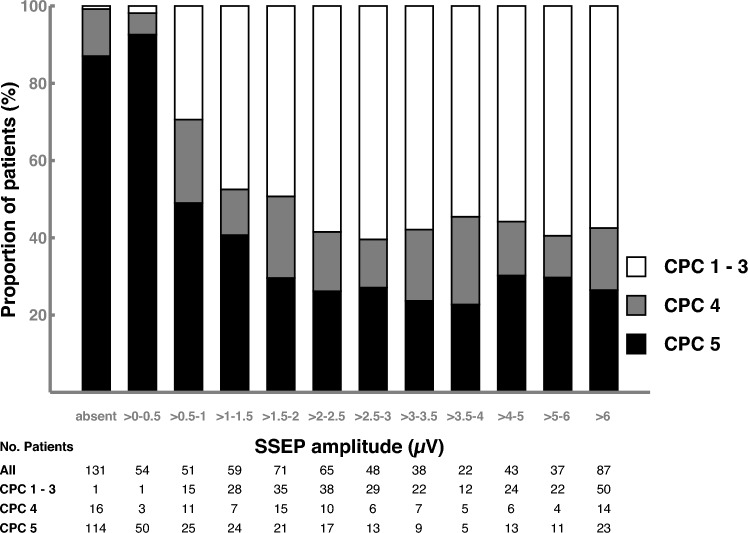
Proportion of clinical outcome stratified by thresholds of cortical SSEP amplitudes. This figure shows the association between proportion of clinical outcome and increase of cortical SSEP amplitude. Clinical outcome is separated into CPC 5 (black bar), CPC 4 (gray bar), and CPC 1–3 (white bar). Absolute patient numbers are provided below the figure. SSEP somatosensory evoked potential, CPC Cerebral performance category

Figure 2 shows the association between clinical outcome and increasing cortical SSEP amplitudes. In 131 patients with bilaterally absent cortical SSEPs, 1 (0.8%) patient had a CPC 1–3, 16 (12.2%) CPC 4, and 114 (87.0%) CPC 5. In 54 patients with cortical SSEP amplitude below 0.5 µV, 1 (1.9%) had a CPC 1 – 3, 3 (5.6%) CPC 4, and 50 (92.6%) CPC 5. In patients with cortical SSEP amplitude above 2.5 µV, the outcome distribution remained largely stable with increasing amplitudes (CPC 1–3: 54.6 – 60.4%, CPC 4: 10.8–18.4%, and CPC 5: 22.7–29.7%).

The positive predictive value to predict good outcome using cortical SSEP amplitudes above 2.5 µV was 57.8% (159/275). 42 (40.4%) of 104 CPC 4 patients had cortical SSEP amplitudes above 2.5 µV of whom 21 patients improved neurologically or died early after ICU discharge with potential death causes other than HIE. Considering improvement after discharge and death causes from other than HIE, the proportion of CPC 4 patients with cortical SSEP above 2.5 µV without identifiable confounders ranged between 0 and 23.6% in the four centers. 22.8% (74/325) of CPC 5 patients had cortical SSEP amplitudes above 2.5 µV.

The lower cortical SSEP amplitude threshold to predict poor outcome did not relevantly change during the first days after CA (Supplementary Fig. 5). Except for one patient with CPC 3 and bilaterally absent SSEPs, the lowest cortical SSEP amplitude of good outcome patients was 0.76 µV, 0.50 µV, 0.63 µV, 0.59 µV, and 0.73 µV, on days 1, 2, 3, 4, and 5, respectively.

### Inter-recording agreement in repeated SSEPs

**Fig. 3:**
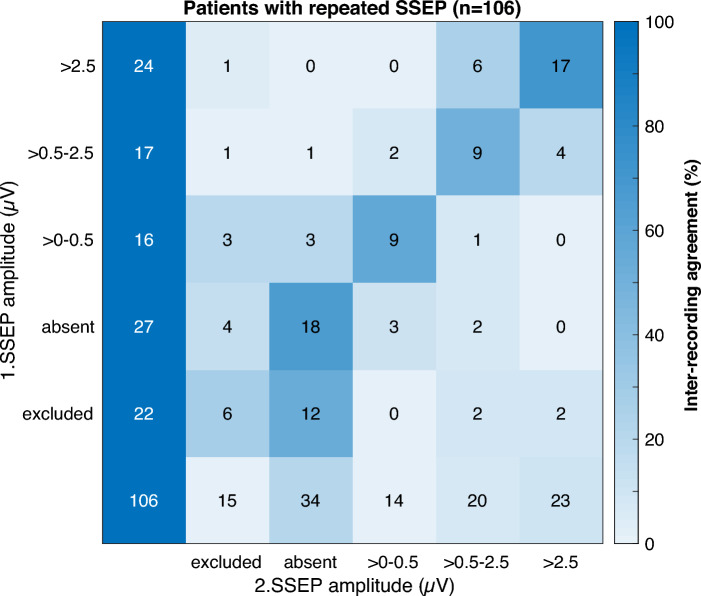
Inter-recording agreement in patients with a repeated SSEP. This heatmap shows the inter-recording agreement on determination of cortical SSEP amplitudes in 106 patients with a repeated SSEP. SSEPs were categorized into excluded due to noise, bilaterally absent, up to 0.5 µV, larger than 0.5–2.5 µV, and above 2.5 µV. The column shows the results from the first SSEP and the row from the repeated SSEP. Numbers in the field represent absolute patient numbers. The inter-recording agreement between first and repeated SSEP is indicated by the field colors in percentages. SSEP somatosensory evoked potential

106 patients had a repeated SSEP at a median of 6 days (4–7) after CA. Figure 3 illustrates changes in cortical SSEP amplitude categories from first to repeated SSEP. 22 (20.8%) were excluded due to high noise levels or bilaterally non-reproducible cervical potentials, 27 (25.5%) had bilaterally absent cortical SSEPs, and 57 (53.8%) had cortical SSEP amplitudes (Supplementary Table 3). 59 (55.7%) patients had no change in SSEP categories. Of 22 excluded patients in the first SSEP, 12 (54.6%) had bilaterally absent cortical SSEPs in the repeated SSEP.

6 (5.7%) patients had prognostication-relevant changes in SSEP categories. In 3 patients with SSEP amplitude decrease, a second CA, refractory cardiogenic shock, and progressive global brain edema were likely reasons. In 3 patients with SSEP amplitude increase, noise levels might have contributed to different cortical SSEP amplitudes between recordings in one patient, apart from the CPC 3 patient with bilaterally absent cortical SSEPs and cortical amplitudes of 0.51 µV in the repeated SSEP.

### Inter-rater agreement on cortical SSEP amplitudes

**Fig. 4:**
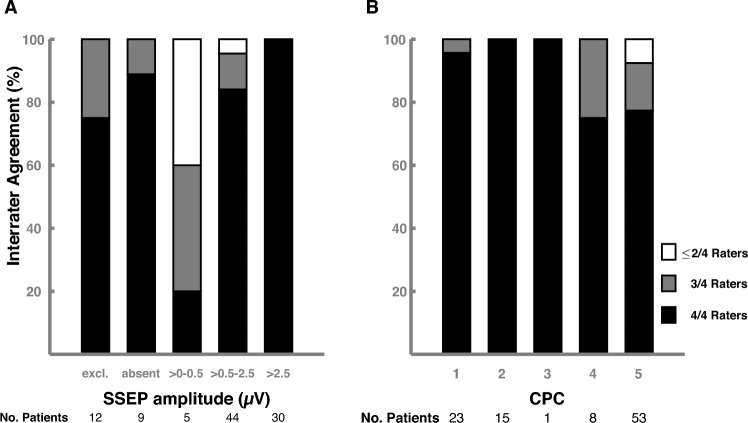
Inter-rater agreement on determination of cortical SSEP amplitudes. This figure illustrates the inter-rater agreement on determination of cortical SSEP amplitudes by four independent raters in 100 patients. **A** SSEPs were categorized into excluded due to noise, bilaterally absent, up to 0.5 µV, larger than 0.5–2.5 µV, and above 2.5 µV. Percentage of agreement between all four raters (black bar), three raters (gray bar), and less or equal two raters (white bar) for the SSEP categories are shown. **B** Percentage of agreement between all four raters (black bar), three raters (gray bar), and less or equal two raters (white) are shown for the clinical outcome groups. SSEP somatosensory evoked potential, excl. excluded, CPC cerebral performance category

Supplementary Table 4 shows the patient flow of the cortical SSEP amplitude stratification by the four raters. Fleiss’ kappa was 0.88 indicating almost perfect inter-rater agreement. In 85 of 100 SSEPs, all four raters agreed on the cortical SSEP category, and at least three of four raters agreed in 96 of 100 SSEPs (Fig. 4A). In 15 patients with inter-rater disagreement (Supplementary Fig. 7), reasons were uncertainty regarding distinction between bilaterally absent and low-amplitude (i.e., 0.1–0.3 µV) cortical SSEPs, interpretation of SSEP with high noise levels, and measuring inaccuracy of cortical SSEP amplitudes ranging from 0.1 to 0.2 µV. Of these 15 patients, 14 had CPC 4–5, and there was no case in which inter-rater disagreement would have led to a different prognostic conclusion (Fig. 4B). Among 400 SSEP ratings, no good outcome patient was erroneously interpreted as to have bilaterally absent or low-amplitude SSEP amplitude.

## Discussion

Our main findings are: (1) bilaterally absent and cortical SSEP amplitudes below 0.5 µV predicted poor outcome with high specificity. (2) We found one patient among 131 patients with bilaterally absent cortical SSEPs, who regained consciousness, but had severe long-term neurological deficits. (3) High-amplitude cortical SSEP amplitudes argued against, but did not exclude severe HIE. (4) Repeated SSEP recordings yielded mostly identical cortical SSEP amplitude categories within the first days after CA. (5) Following a standardized evaluation pathway, inter-rater agreement on cortical SSEP amplitudes was high.

Cortical SSEP amplitude are generated by the summation of postsynaptic potentials in the somatosensory cortex [23, 27–29]; hence, it is plausible that HIE decreases the number of intact neurons generating lower cortical SSEP amplitudes. The histopathological association of decreased cortical SSEP amplitudes and increased neuronal death has been previously studied [25].

In our study, bilaterally absent cortical SSEP predicted poor outcome with high specificity of 99.2% corroborating previous studies [5–7, 17, 30–32]. Importantly, neuroprognostication studies are prone to a self-fulfilling prophecy if prognostic tests influence WLST decisions. The prognostic reliability of bilaterally absent cortical SSEP has been previously questioned [13]. However, reevaluation of cases with good outcome despite SSEPs interpreted as bilaterally absent [3], studies from countries with limited WLST [31, 33, 34], postmortem histological analyses [25, 35], and follow-ups on CPC 4 patients [24] confirmed the prognostic reliability of bilaterally absent cortical SSEP.

In our current study, one 18-year-old patient with initially bilaterally absent cortical SSEPs regained consciousness, but suffered from severe long-term neurological deficits. This illustrates that bilaterally absent SSEPs do not preclude recovery of consciousness with absolute certainty [36–38]. Rare cases of recovery of consciousness despite bilaterally absent SSEP and the observation of erroneous interpretation of SSEP recordings in clinical routine underscore the importance of multimodal neuroprognostication and considering confounders as recommended in the recent international guidelines [1, 2].

Except for this case, the lowest cortical SSEP amplitude in good outcome was 0.5 µV, and patients without regain of consciousness had a median of 0.7 µV. This is in line with several studies [5–11], which found a lower cortical SSEP amplitude threshold ranging from 0.45 to 1.0 µV in good outcome. Using a threshold of 0.5 µV instead of bilaterally absent cortical SSEP, the sensitivity to predict poor outcome increased from 30.3 to 42.9% in our study, compared to 47–86% in other studies [6–9, 11, 33, 39]. Sensitivities depend on cohort characteristics for SSEP as shown by the differencing amplitude distributions across the four centers.

We assessed clinical outcome upon ICU discharge. Hence, we cannot exclude that CPC 4 patients with bilaterally absent or low-amplitude cortical SSEP amplitudes improved neurologically in long-term. The median duration of the ICU stay of CPC 4 patients was 23 and 29 days in center 1 and 2. We have previously shown that long-term improvement in CPC 4 patients beyond this observation period on the ICU is rare and was not observed among patients with poor early prognostic testing such as bilaterally absent SSEP [24]. Other studies on late awakening after CA also support this time threshold [40, 41].

Importantly, adherence to international guidelines [22, 23] and electrode positions [42] are mandatory as subtle changes can lead to differences in cortical SSEP amplitudes. Furthermore, interindividual differences of head configurations may cause differences in cortical SSEP amplitudes despite precise electrode placing. Hence, an amplitude safety margin in low-amplitude cortical SSEPs is reasonable. Compared to EEG, sedation has substantially less effects on cortical SSEP amplitudes [6, 43, 44]. Early recording during hypothermia did not decrease the specificity to predict poor outcome [34], and recording timing had no effect on cortical SSEP amplitudes in our cohort and a previous study [6].

Finding prognostic tests to predict the absence of severe HIE is difficult. We previously found that high cortical SSEP amplitudes indicated absence of severe HIE [6, 25] at a threshold of 2.5–2.7 µV, which was validated by the amplitudes of CPC 4 patients in other cohorts [5, 7, 10, 24]. In our study, 42 of 104 CPC 4 patients had cortical SSEP amplitudes above 2.5 µV. 21 patients neurologically improved or died early after ICU discharge with potential causes of death other than HIE. Importantly, 32 (76.2%) of those CPC 4 patients were included from one center which had the shortest ICU durations. These patients might have regained consciousness upon longer follow-up. In contrast, only nine patients with CPC 4 from center 1 and 2 had amplitudes larger than 2.5 µV. While good outcome clinically proves absence of severe HIE, deceased patients may or may not have severe HIE, as extracerebral complications frequently cause death despite no HIE [32, 45]. In our study, regain of consciousness was associated with significantly higher cortical SSEP amplitudes compared to patients never regaining consciousness. Hence, cortical SSEP amplitudes above 2.5 µV argue against, but do not exclude severe HIE, and should prompt further prognostic testing if neuroprognostication suggest otherwise.

Two previous studies found moderate to substantial inter-rater agreement on classifying cortical SSEPs as bilaterally present or absent with noise as main confounder of disagreement [15, 16]. Insufficient noise levels, non-adherence to guidelines, and failure to demonstrate intact peripheral and spinal conduction can cause false interpretation of SSEPs [3, 13, 14] with particular risk to falsely classify low-amplitude cortical SSEPs as bilaterally absent [17].

In our study, four raters with different expertise achieved almost perfect inter-rater agreement following a standardized SSEP evaluation pathway with a tolerable noise level of 0.25 µV. Our Fleiss’ kappa of 0.88 was higher compared to 0.34–0.75 in previous studies [15, 16], which only classified SSEPs as present or bilaterally absent. Hence, we argue that determining amplitudes of cortical SSEPs is safer and meets higher inter-rater agreement than dichotomizing SSEP into present or bilaterally absent. However, prerequisites are a definition of acceptable cortical noise level, strict adherence to a standardized evaluation pathway, and sufficient training by an experienced supervisor. Insufficient noise levels should be recognized and improved during recordings, e.g., using short-acting muscle relaxants [14] and/or by repeating SSEPs. These prerequisites are even more important considering that inter-rater disagreement nearly exclusively occurred in CPC 4–5 patients in our study. However, in none of these cases neuroprognostication would have changed if we had only relied on cortical SSEP amplitudes as a single prognostic test. Our results should be replicated by future studies; hence, we published our SSEP pathway with the training set of SSEP examples including the 15 instructive SSEP cases with inter-rater disagreement.

Prognostic results from repeated SSEPs were shown to be stable between recordings within the first days after CA [34, 46]. Corroborating these results there were only 6 (5.7%) patients with neuroprognostication-relevant changes of cortical SSEP amplitudes with three days between recordings. Secondary complications likely contributed to decrease and noise level to increase of cortical SSEP amplitudes. Our good inter-recording agreement validated the reliability of cortical SSEP amplitude between recordings and argues against routinely repeating SSEP in low-noise recordings.

Our study has strengths and limitations. Strengths include the large sample size, four participating centers with different recording devices, recording parameters, and cohorts, blinded SSEP amplitude assessment using a standardized SSEP evaluation pathway, blinded evaluation of inter-rater agreement, and a large number of repeated SSEPs. Altogether, these features support generalizability of our results.

Due to the retrospective and multicenter design, our study cohort was heterogenous. However, the main results remained unchanged in the subgroup analyses, and we could study the effect of patient selection on cortical SSEP amplitudes. We assessed clinical outcome upon ICU discharge and, due to legal restrictions, could not routinely obtain long-term follow-up. Importantly, CA patients may substantially recovery during rehabilitation [47–49]. Therefore, we classified CPC 3 as good outcome. Compared to previous studies, we included a higher proportion of CPC 4 patients due to center characteristics. We cannot exclude long-term improvement of CPC 4 patients, especially for patients recruited in center 4 with shorter ICU stays. As most other neuroprognostication studies, we cannot exclude a self-fulfilling prophecy for SSEPs. Prognostication at the participating centers is performed with great caution using a multimodal prognostic approach and with a considerable clinical observation period in unclear cases.

In summary, in this retrospective multicenter study of 706 comatose patients with SSEPs after CA, bilaterally absent and cortical SSEP amplitudes below 0.5 µV predicted poor outcome with high specificity. One young patient with bilaterally absent cortical SSEPs regained consciousness with severe long-term neurological deficits. High-amplitude cortical SSEP amplitudes likely indicate absence of severe HIE. Using a standardized evaluation pathway, inter-recording agreement and inter-rater agreement were reliable to routinely use cortical SSEP amplitudes in multimodal neuroprognostication.

### Supplementary Information

Below is the link to the electronic supplementary material.

Supplementary file1 (PDF 5318 KB)

## Data Availability

Anonymized data are available on reasonable request to the corresponding author.

## Abbreviations

SSEP: Somatosensory evoked potentials
CA: Cardiac arrest
CPC: Cerebral performance category
IQR: Inter-quartile range
HIE: Hypoxic-ischemic encephalopathy
WLST: Withdrawal of life-sustaining therapy
ICU: Intensive care unit
