# Supplementary material for: Cortical somatosensory evoked potential amplitudes and clinical outcome after cardiac arrest: a retrospective multicenter study
Source: J Neurol. 2023 Aug 28;270(12):5999–6009. doi: 10.1007/s00415-023-11951-4 (PMC10632270; doi:10.1007/s00415-023-11951-4)
Supplement: Supplementary file 1 — Supplementary file1 (PDF 5318 KB) [file 415_2023_11951_MOESM1_ESM.pdf]

Supplementary Material

Cortical somatosensory evoked potential amplitudes and clinical outcome after cardiac arrest: A retrospective multicenter study

Noelle Aalberts, MD, Erik Westhall, PhD, Birger Johnsen, PhD, Katrin Hahn, MD, Martin Kenda, MD, Tobias Cronberg, PhD, Hans Friberg, PhD, Sandra Preuß, MD, Christoph J. Ploner, MD, Christian Storm, MD, Jens Nee, MD, Christoph Leithner, MD, Christian Endisch, MD

Table of Contents

Supplementary Figure 1. Pathway 1 for evaluation of SSEPs based on noise level and technical preconditions. **p 3**

Supplementary Figure 2. Pathway 2 for determination of cortical SSEP amplitudes. **p 4**

Supplementary Table 1. Technical details of SSEP recording stratified by the four centers. **p 5**

Supplementary Table 2. Patient flow for the first SSEP. **p 6**

Supplementary Table 3. Patient flow for the repeated SSEP. **p 7**

Supplementary Figure 3. Cortical SSEP amplitudes and clinical outcome stratified by the four centers. **p 8**

Supplementary Figure 4. Association between cortical SSEP amplitudes and regain of consciousness. **p 9**

Supplementary Figure 5. Cortical SSEP amplitudes and recording timing. **p 10**

Supplementary Figure 6. SSEP examples for supervised training. **p 11 – 12**

Supplementary Table 4. Patient flow of determination of cortical SSEP amplitude stratified by the four raters. **p 13**

Supplementary Figure 7. SSEP cases with interrater disagreement. **p 14**

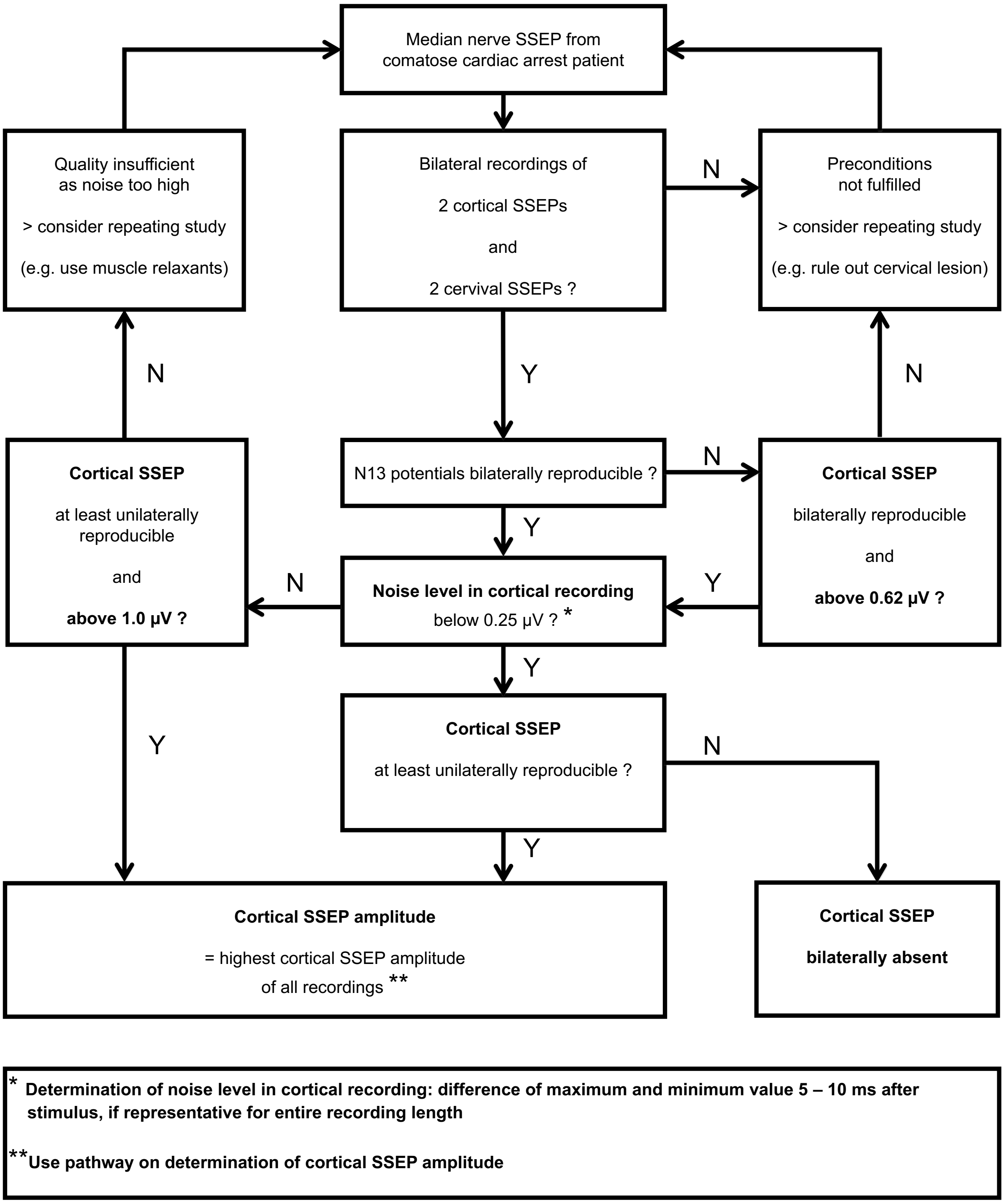

Supplementary Figure 1. Pathway 1 for evaluation of SSEPs based on noise level and technical preconditions.

We previously established this pathway and evaluated the SSEPs according to an adapted version. Importantly, the cortical SSEP amplitude threshold of 0.62 µV was chosen as this was the lowest cortical SSEP amplitude in good outcome patients in Endisch et al. 2015 (doi.org/10.1212/wnl.0000000000002123). Our pragmatic pathway hereby follows recent guidelines of the International Federation of Clinical Neurophysiology and American Clinical Neurophysiology Society. SSEP – somatosensory evoked potential.

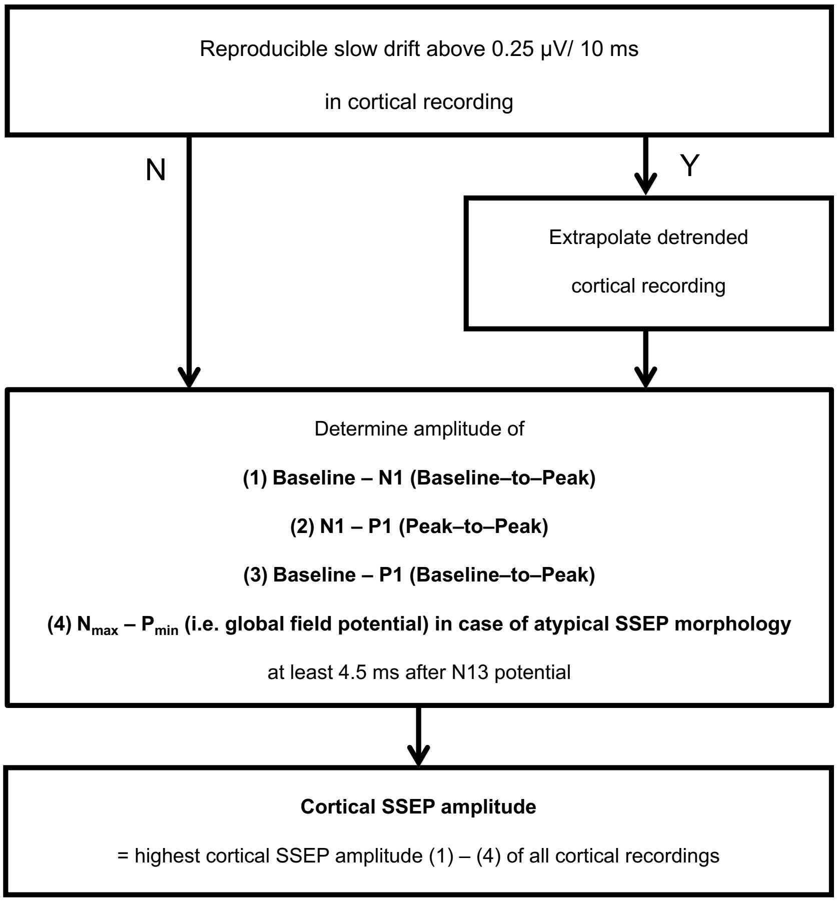

Supplementary Figure 2. Pathway 2 for determination of cortical SSEP amplitudes.

In accordance to our pathway 1, this pathway was established in Endisch et al. 2015 and used in the present study to determine cortical SSEP amplitudes. N1 is the first negative peak of the cortical SSEP and P1 the first positive peak. N_{max} is the maximum value and P_{min} the minimum value of the cortical SSEP recording 4.5 ms after the cervival N13 potential and within 50 ms. SSEP – somatosensory evoked potential. See doi.org/10.1212/wnl.0000000000002123 for the reference of Endisch et al. 2015.

Technical parameter	Center 1	Center 2	Center 3	Center 4
**Indication of SSEP after cardiac arrest**	Comatose after rewarming	Comatose after rewarming	Comatose after rewarming, SSEP usually as final prognostic test	Comatose after rewarming
**Cortical lead**	CP3/CP4 – Fz	CP3/CP4 – Fz	CP3/CP4 – Fz	CP3/CP4 – Fz
**Impulse form**	Monophasic square-wave pulse	Monophasic square-wave pulse	Monophasic square-wave pulse	Monophasic square-wave pulse
**Pulse width, µs**	200	200	200	200
**Stimulation rate, Hz**	2.7	3.0	3.1	3.0
**Stimulation intensity**	Visible thumb twitch	Visible thumb twitch	Visible thumb twitch	Visible thumb twitch
**Contact impedance, kOhm**	below 5	below 5	below 5	below 5
**Bandwidth, Hz**	5 – 1500	20 – 3000	30 – 3000	10 – 3000
**Recording time, ms**	50	50	50	100
**Averaged repetitions, n**	500	500	500	500
**Replications, n**	at least 2	at least 2	at least 2	at least 2

Supplementary Table 1. Technical details of SSEP recording stratified by the four centers.

This table provides the technical SSEP recordings details of each center. Center 1: Charité University Hospital, Berlin, Germany, Campus Virchow-Klinikum; Center 2: Charité University Hospital, Berlin, Germany, Campus Mitte; Center 3: Skåne University Hospital, Lund, Sweden; and Center 4: Aarhus University Hospital, Aarhus, Denmark. SSEP – somatosensory evoked potential.

	All	Center 1	Center 2	Center 3	Center 4
Enrolled patients (n, %)	816 (100)	458 (56.1)	67 (8.2)	103 (12.6)	188 (23.0)
Excluded due to incomplete recordings (n, %)	9 (1.1)	5 (1.1)	2 (3.0)	2 (1.9)	0 (0)
N13 not bilaterally reproducible and cortical SSEP amplitude < 0.62 µV (n, %)	48 (5.9)	22 (4.8)	6 (9.0)	12 (11.7)	8 (4.3)
N13 not bilaterally reproducible, noise < 0.25 µV and cortical SSEP amplitude > 0.62 µV (n, %)	6 (0.7)	3 (0.7)	0 (0)	3 (2.9)	0 (0)
N13 not bilaterally reproducible, noise > 0.25 µV and cortical SSEP amplitude > 1.0 µV (n, %)	20 (2.5)	11 (2.4)	6 (9.0)	0 (0)	3 (1.6)
Noise < 0.25 µV and bilaterally absent cortical SSEP (n, %)	131 (16.1)	63 (13.8)	12 (17.9)	37 (35.9)	19 (10.1)
Noise < 0.25 µV and cortical SSEP (n, %)	439 (53.8)	270 (59.0)	29 (43.3)	44 (42.7)	96 (51.1)
Noise > 0.25 µV and cortical SSEP amplitude > 1.0 µV (n, %)	110 (13.5)	73 (15.9)	10 (14.9)	1 (1.0)	26 (13.8)
Excluded due to noise > 0.25 µV (n, %)	53 (6.5)	11 (2.4)	2 (3.0)	4 (3.9)	36 (19.2)
Included patients (n, %)	706 (86.5)	420 (91.7)	57 (85.1)	85 (82.5)	144 (76.6)

Supplementary Table 2. Patient flow for the first SSEP.

For the first SSEP, this table shows the flow of enrolled patients according to the two pathways on noise level, technical preconditions, and determination of cortical SSEP amplitudes. Results are stratified by the four centers. Center 1: Charité University Hospital, Berlin, Germany, Campus Virchow-Klinikum; Center 2: Charité University Hospital, Berlin, Germany, Campus Mitte; Center 3: Skåne University Hospital, Lund, Sweden; and Center 4: Aarhus University Hospital, Aarhus, Denmark. SSEP – somatosensory evoked potential.

	All	Center 1	Center 2	Center 3	Center 4
Enrolled patients (n, %)	106 (100)	62 (58.5)	12 (11.3)	4 (3.8)	28 (26.4)
Excluded due to incomplete recordings (n, %)	0 (0)	0 (0)	0 (0)	0 (0)	0 (0)
N13 not bilaterally reproducible and cortical SSEP amplitude < 0.62 µV (n, %)	9 (8.5)	1 (1.6)	4 (33.3)	0 (0)	4 (14.3)
N13 not bilaterally reproducible, noise < 0.25 µV and cortical SSEP amplitude > 0.62 µV (n, %)	1 (0.9)	1 (1.6)	0 (0)	0 (0)	0 (0)
N13 not bilaterally reproducible, noise > 0.25 µV and cortical SSEP amplitude > 1.0 µV (n, %)	1 (0.9)	0 (0)	1 (8.3)	0 (0)	0 (0)
Noise < 0.25 µV and bilaterally absent cortical SSEP (n, %)	34 (32.1)	28 (45.2)	2 (16.7)	2 (50)	2 (7.1)
Noise < 0.25 µV and cortical SSEP (n, %)	46 (43.4)	23 (37.1)	4 (33.3)	2 (50)	17 (60.7)
Noise > 0.25 µV and cortical SSEP amplitude > 1.0 µV (n, %)	9 (8.5)	4 (6.5)	1 (8.3)	0 (0)	4 (14.3)
Excluded due to noise > 0.25 µV (n, %)	6 (5.7)	5 (8.1)	0 (0)	0 (0)	1 (3.6)
Included patients (n, %)	91 (85.9)	56 (90.3)	8 (66.7)	4 (100)	23 (82.1)

Supplementary Table 3. Patient flow for the repeated SSEP.

For the repeated SSEP, this table shows the flow of enrolled patients according to the two pathways on noise level, technical preconditions, and determination of cortical SSEP amplitudes. Results are stratified by the four centers. Center 1: Charité University Hospital, Berlin, Germany, Campus Virchow-Klinikum; Center 2: Charité University Hospital, Berlin, Germany, Campus Mitte; Center 3: Skåne University Hospital, Lund, Sweden; and Center 4: Aarhus University Hospital, Aarhus, Denmark. SSEP – somatosensory evoked potential.

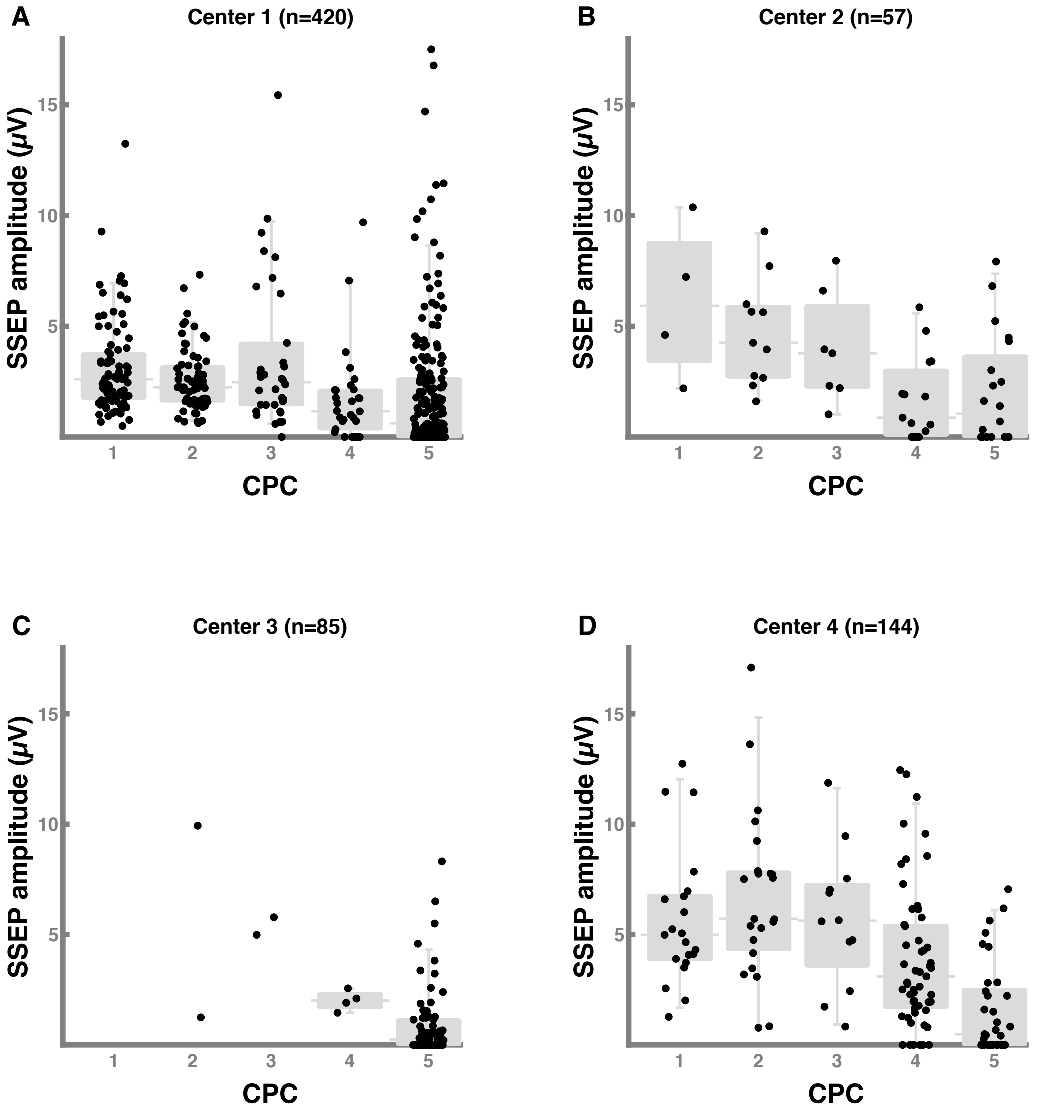

Supplementary Figure 3. Cortical SSEP amplitudes and clinical outcome stratified by the four centers.

The subplots show the association between cortical SSEP amplitudes and clinical outcome assessed. Scatterplots (black dots) show raw data and box plots (grey) interquartile range, median, and whisker bars representing the 5th and 95th percentile. (A) Center 1: Charité University Hospital, Berlin, Germany, Campus Virchow-Klinikum. CPC 5 patients had a median cortical SSEP amplitude of 0.6 µV (0 – 2.6), which was lower compared to CPC 1 and CPC 3 patients (median 2.6 µV, 1.7 – 3.8, and 2.5 µV, 1.4 – 4.2, respectively). (B) Center 2: Charité University Hospital, Berlin, Germany, Campus Mitte. CPC 5 patients had a median cortical SSEP amplitude of 1.0 µV (0 – 3.7), which was lower compared to CPC 1 and CPC 2 patients (median 5.9 µV, 3.4 – 8.8, and 4.3 µV, 2.7 – 5.9, respectively). (C) Center 3: Skåne University Hospital, Lund, Sweden. CPC 5 patients had a median cortical SSEP amplitude of 0.25 µV (0 – 1.1), which was lower compared to CPC 2 and CPC 3 patients (median 5.6 µV and 5.4 µV, respectively). (D) Center 4: Aarhus University Hospital, Aarhus, Denmark. CPC 5 patients had a median cortical SSEP amplitude of 0.49 µV (0 – 2.5), which was lower compared to all other four CPC groups. SSEP – somatosensory evoked potential, CPC – Cerebral performance category.

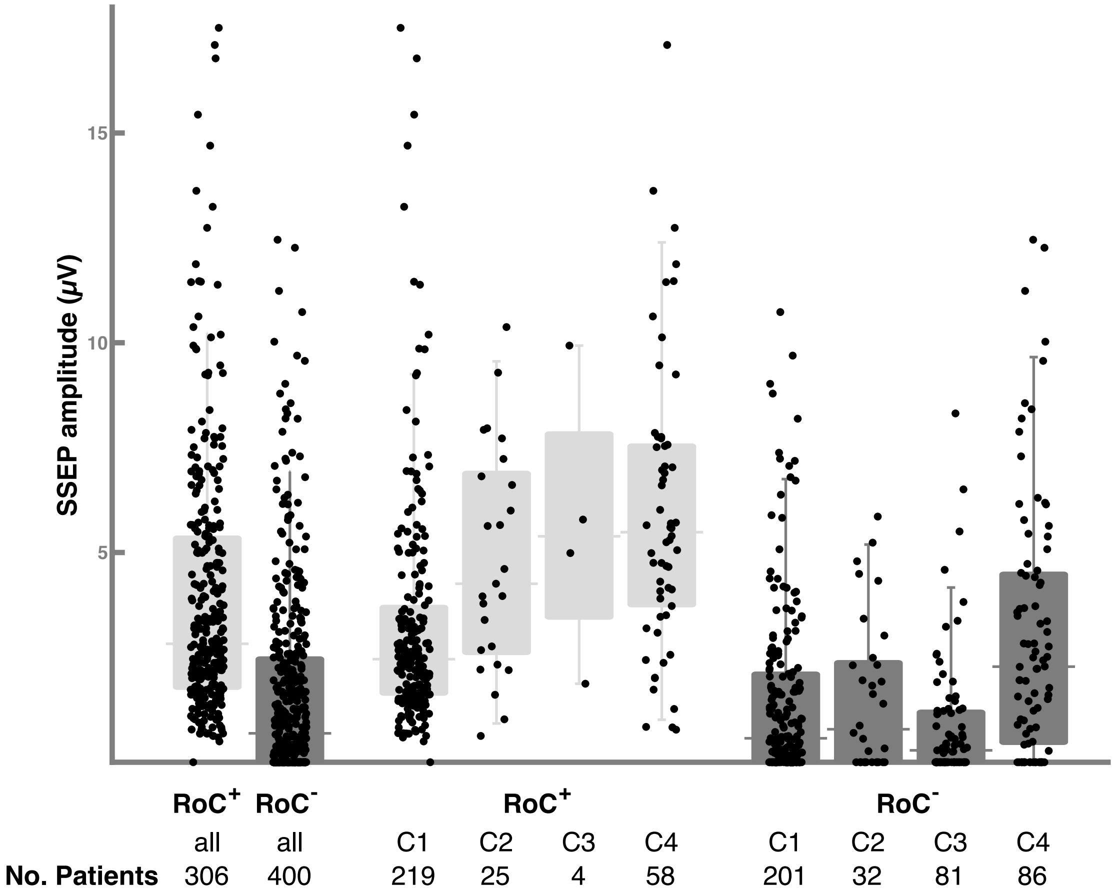

Supplementary Figure 4. Association between cortical SSEP amplitudes and regain of consciousness.

This figure shows the cortical SSEP amplitudes dependent on whether or not patients regained consciousness during the intensive care unit stay. The results are presented as scatterplots (black dots) and box plots (grey) with interquartile range, median, and whisker bars representing the 5th and 95th percentile. We studied the association for all included patients and centers. Absolute patient numbers are provided below the figure. SSEP – somatosensory evoked potential, RoC^{+} – regain of consciousness present, RoC^{-} – no regain of consciousness. C1 – center 1, C2 – center 2, C3 – center 3, C4 – center 4. Center 1: Charité University Hospital, Berlin, Germany, Campus Virchow-Klinikum; Center 2: Charité University Hospital, Berlin, Germany, Campus Mitte; Center 3: Skåne University Hospital, Lund, Sweden; and Center 4: Aarhus University Hospital, Aarhus, Denmark.

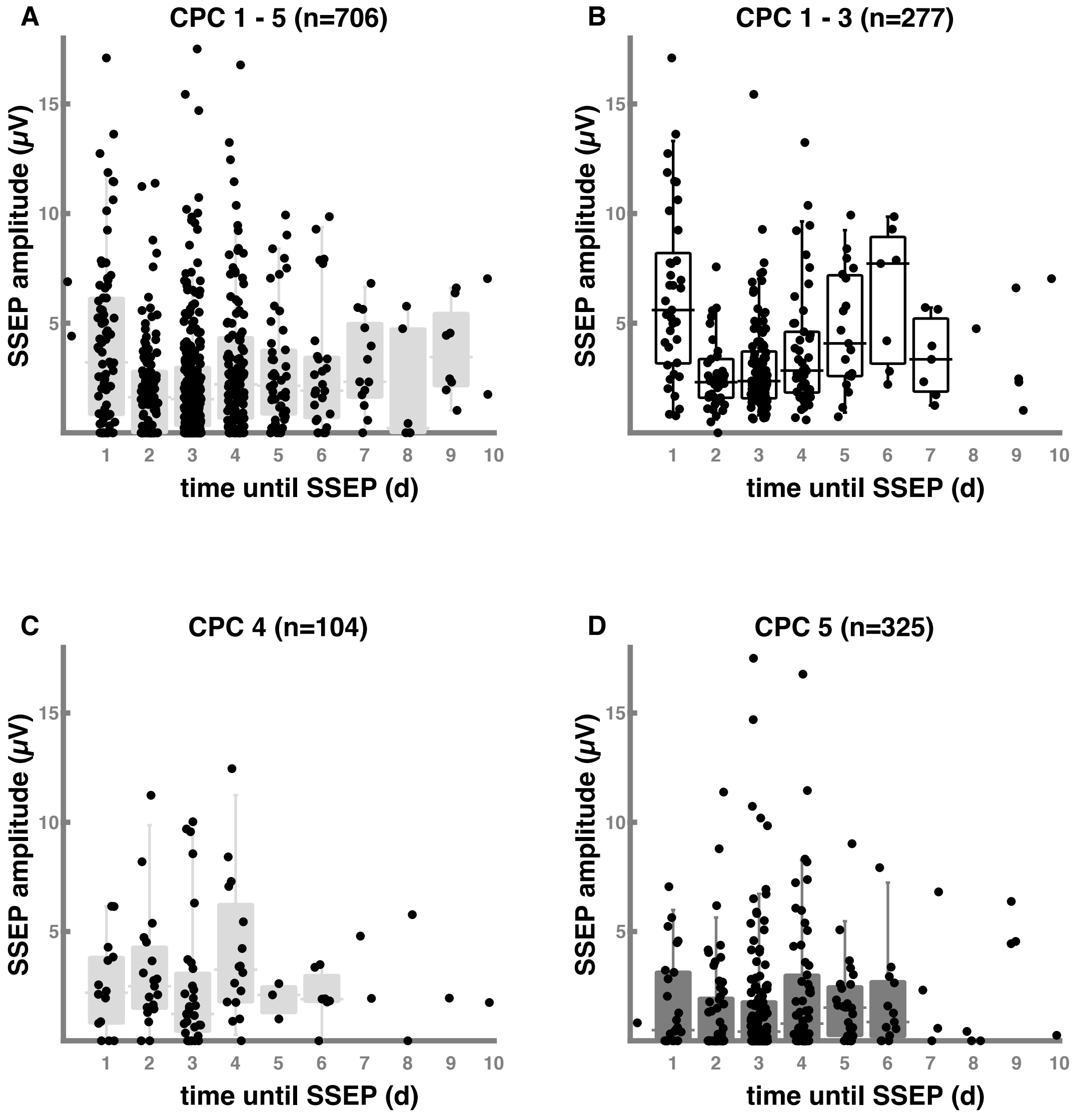

Supplementary Figure 5. Cortical SSEP amplitudes and recording timing.

This figure illustrates the association between cortical SSEP amplitudes and day of recording for different clinical outcomes. We present the data as scatterplots (black dots) and box plots (grey) with interquartile range, median, and whisker bars representing the 5th and 95th percentile as appropriated. (A) All included patients. (B) Patients with a CPC outcome of 1 – 3. (C) CPC 4 patients. (D) CPC 5 patients. SSEP – somatosensory evoked potential, CPC – Cerebral performance category.

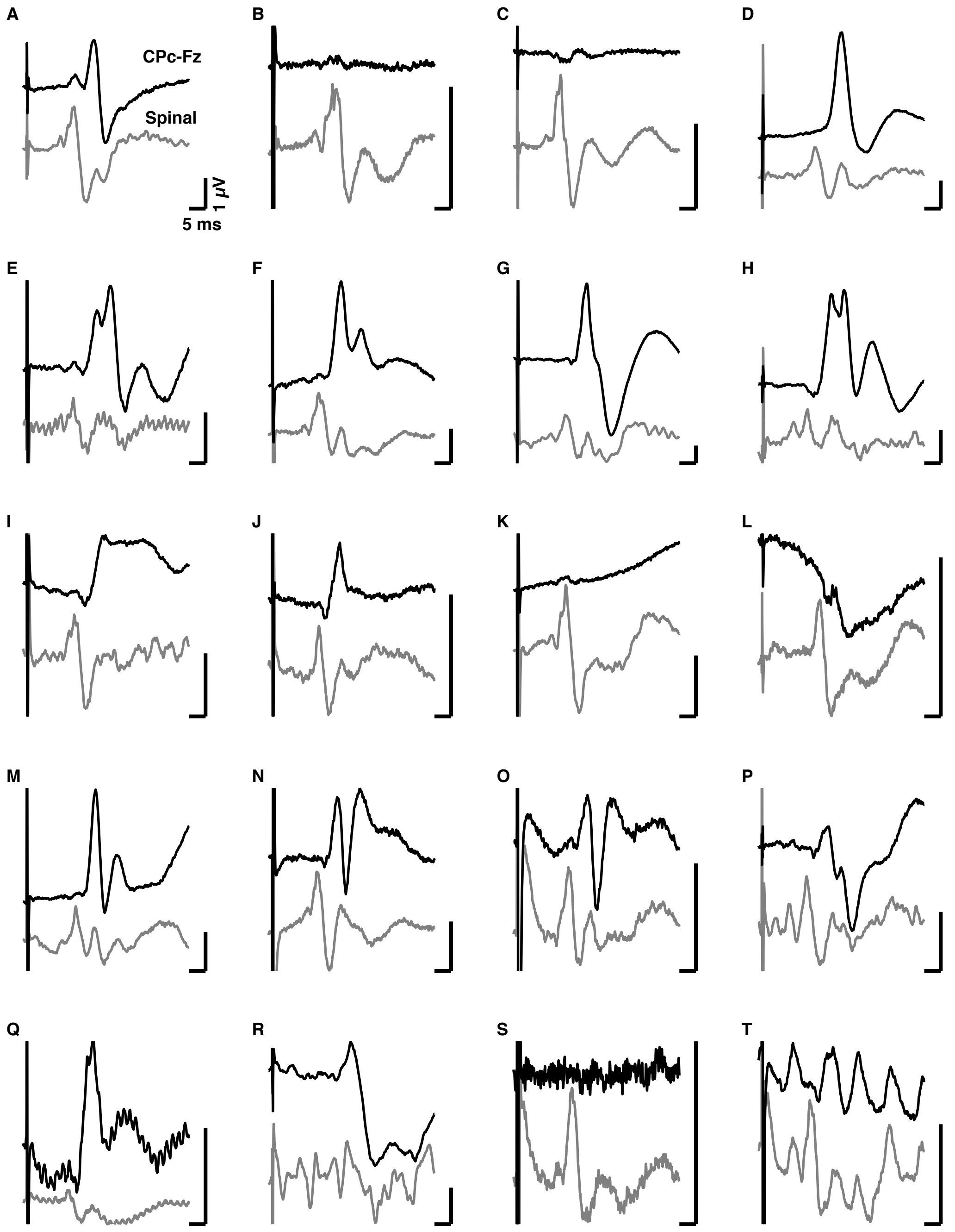

Supplementary Figure 6. SSEP examples for supervised training.

This figure shows the 20 exemplary SSEPs of the supervised training for the interrater analysis. Each subfigure shows a cortical (black) and a cervical (grey) recording. For interest of clarity, we show only one cortical and one cervical recording for each patient. (A) Typical middle-amplitude SSEP with N1-P1 configuration. (B) Absent cortical SSEP with tolerable noise. (C) Low-amplitude SSEP with tolerable noise. (D) High-amplitude SSEP. (E – H) Double peaked SSEPs with different peak configurations. (I) SSEP with Baseline-N1 configuration without return to baseline. (J) SSEP with Baseline-N1 configuration with nearly return to baseline. (K) Low-frequency noise with slow trend, but tolerable noise to classify as absent cortical SSEP. (L) Low-frequency noise with

discernible low-amplitude SSEP. (M – 0) Double peaked SSEPs with deep P1 with different configurations. (P) Rare atypical SSEP configuration with $N_{max} – P_{min}$ as the highest amplitude. (Q) Sufficiently discernible cortical SSEP despite overlapping high-frequency noise. (R) Example of a patient who had no reproducible cervical N13 but cortical SSEP amplitudes above 1.0 μV. (S) Absent cortical SSEP with excellent noise level. Note the zoomed y-axis. (T) Recording that was excluded due to insufficient noise level.

	Rater 1 (C.E.)	Rater 2 (C.L.)	Rater 3 (M.K.)	Rater 4 (N.A.)
Enrolled patients (n, %)	100 (100)	100 (100)	100 (100)	100 (100)
Excluded due to incomplete recordings (n, %)	2 (2)	2 (2)	2 (2)	2 (2)
N13 not bilaterally reproducible and cortical SSEP amplitude < 0.62 μV (n, %)	5 (5)	5 (5)	5 (5)	3 (3)
N13 not bilaterally reproducible, noise < 0.25 μV and cortical SSEP amplitude > 0.62 μV (n, %)	2 (2)	3 (3)	2 (2)	2 (2)
N13 not bilaterally reproducible, noise > 0.25 μV and cortical SSEP amplitude > 1.0 μV (n, %)	1 (1)	2 (2)	3 (3)	1 (1)
Noise < 0.25 μV and bilaterally absent cortical SSEP (n, %)	9 (9)	12 (12)	12 (12)	10 (10)
Noise < 0.25 μV and cortical SSEP (n, %)	57 (57)	52 (52)	53 (53)	58 (58)
Noise > 0.25 μV and cortical SSEP amplitude > 1.0 μV (n, %)	19 (19)	16 (16)	18 (18)	19 (19)
Excluded due to noise > 0.25 μV (n, %)	5 (5)	8 (8)	4 (4)	5 (5)
Included patients (n, %)	88 (88)	85 (85)	88 (88)	90 (90)

Supplementary Table 4. Patient flow of determination of cortical SSEP amplitude stratified by the four raters.

For each rater, this table shows the flow of unsupervised and independent determination of cortical SSEP amplitude according to the two pathways on noise level, technical preconditions and determination of cortical. SSEP – somatosensory evoked potential.

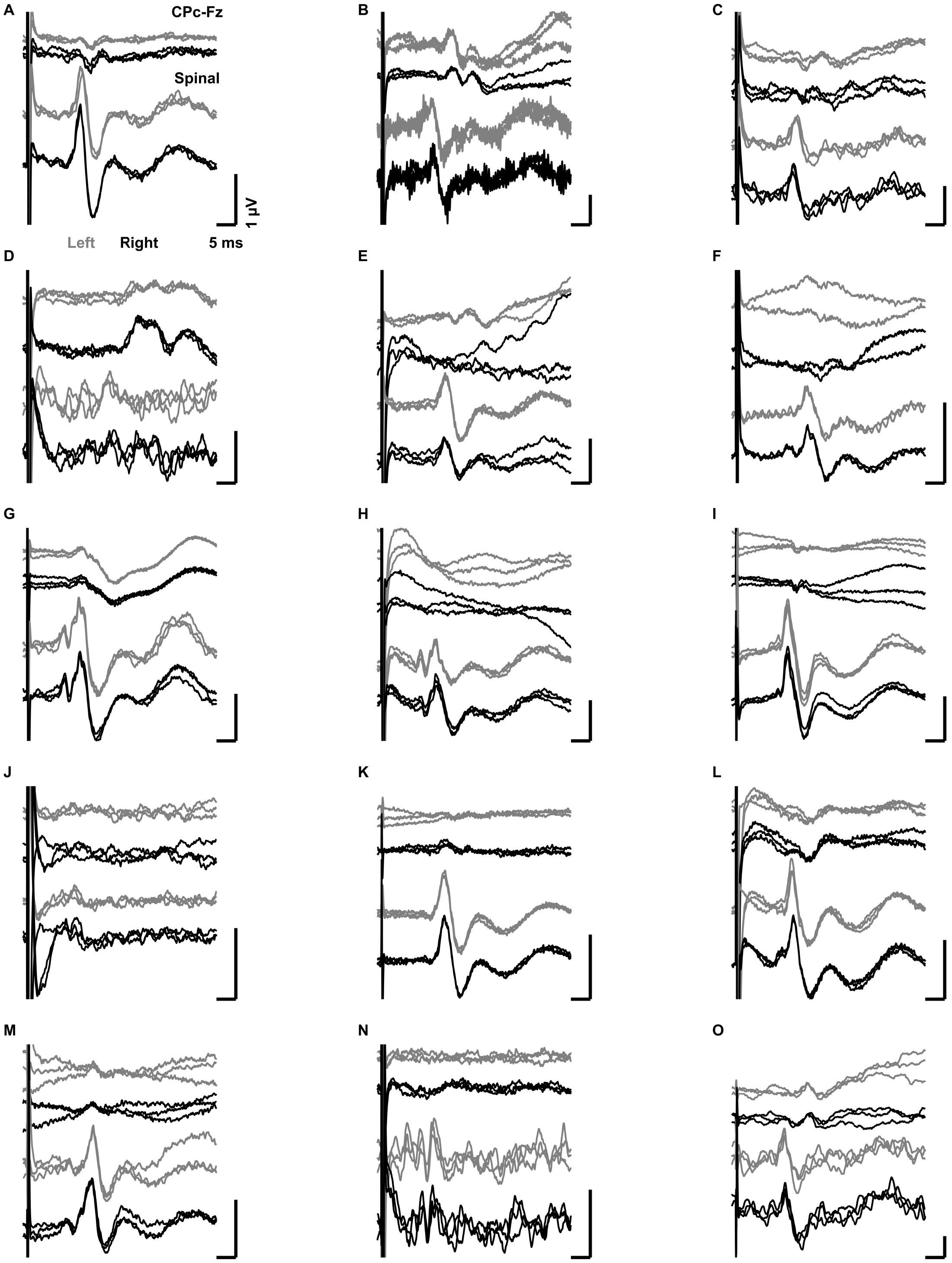

Supplementary Figure 7. SSEP cases with interrater disagreement.

Of 100 SSEPs included in the interrater analysis, this figure illustrates the 15 SSEPs, in which the four raters had disagreement. Each subfigure shows all cortical recordings and cervical recordings from both sides (grey: left and black: right). For interest of clarity, the right scale line (i.e. 5 ms and 1 µV) was only labeled for the first subfigure. Major reasons of interrater disagreement were differentiation between bilaterally absent and very low-amplitude cortical SSEPs (e.g. K), interpretation of SSEPs in high noise levels (e.g. J and N), and SSEP amplitude measuring inaccuracy (e.g. L with two raters measuring 0.57 µV and 0.47 µV, respectively). In none of the 15 patients, a SSEP was classified as bilaterally absent or low-amplitude cortical SSEP in a patient with good outcome.
